# Mental health law in the community: thinking about Africa

**DOI:** 10.1186/1752-4458-5-21

**Published:** 2011-09-13

**Authors:** Peter Bartlett, Rachel Jenkins, David Kiima

**Affiliations:** 1 School of Law and Institute of Mental Health, University of Nottingham, NG7 2RD, Nottingham, UK; 2 King's College London, Institute of Psychiatry, London, UK; 3 Director of Mental Health, Ministry of Medical Services, Kenya, Africa

## Abstract

The new United Nations Convention on the Rights of Persons with Disabilities creates a new paradigm for mental health law, moving from a focus on institutional care to a focus on community-based services and treatment. This article considers implementation of this approach in Africa.

## Introduction

Traditionally, mental health law at both domestic and international levels has focused on institutional care, and particularly psychiatric hospitalisation. In this vision, the role of mental health law has been to ensure appropriate substantive and procedural standards prior to involuntary admission, and, more recently, to ensure standards of institutional care following admission.

Historically, this approach to legislation corresponded to the policies regarding the psychiatric care of people with relatively severe mental illness, which had a central focus on detention in psychiatric asylums, often for extended periods. This paradigm of mental health law can however be seen as increasingly insufficient. The political emphasis in recent decades has moved from institutional to community care for people with relatively severe mental illness, and this shift is reflected in the new UN Convention on the Rights of Persons with Disabilities (CRPD). Unlike many previous international documents such as the UN Mental Illness Principles, the CRPD is not mere guidance: it is international law, with a formal review body to which countries that have signed the convention will be held accountable. The CRPD moves the focus of law away from detention and compulsion, to the provision of community services and the right of a person with disabilities (a term which expressly includes mental disabilities) to integration into the community. Clearly, a legislative focus on institutionalisation to the exclusion of community life is now out of step with the developing international law.

Africa presents particular opportunities and challenges for this new legal paradigm. Its rates of institutionalisation tend to be very low by international standards: see additional file 1, table 1. In rich countries the move from an institutional model of care to a community model of care has been achieved through the development of decentralised community-based dedicated mental health care alternatives provided by specialist professionals in liaison with a strong primary care infrastructure. In 1980 there was around 1 psychiatrist per 100,000 population in the UK; by 1990 this had increased to 1 per 50,000 and by 2010 this ratio is around 1 psychiatrist per 10,000. However, large-scale specialist mental health care provision is not a practical general model for Africa, where per capita GDP is often less than US$ 2000 per year, and where consultant psychiatrists, psychologists, psychiatric nurses, and social workers are strictly limited (see further table 1). A little specialist community provision is often found in the close neighbourhood of psychiatric provincial and district units, practised by enthusiastic specialists who devote some time to following up clients in the community-however, logistically such a specialist delivered community service can only cover a tiny fraction of those in need. Some African countries eg Tanzania, Kenya, Malawi, Zambia are making systematic efforts to integrate mental health into primary care settings with support and supervision supplied by district level mental health staff, who where they exist, tend to be psychiatric nurses [1,2]. Such approaches make major logistical sense in the context of only 1 psychiatric nurse per 250,000 population, 1 psychiatrist per million population, and often no psychiatric social workers or psychologists.

This paper looks at what the new paradigm of law might look like in African contexts. It deliberately does not focus on the regulation of institutions. Such institutions do of course exist in some African countries, generally in the capital cities, and the practices and standards concerning admission to them and care within them raise important human rights issues. A focus on community provision across the country as a whole, whether through primary care or some other method, must not result in the people admitted to these institutions being forgotten or ignored, but such concerns have already attracted some international attention. This article focuses instead on the specific question of what mental health law looks like if it focuses outside the institution, and instead on community and primary care services. While the new legislative paradigm enshrined in the CRPD makes this a relevant question internationally, this paper couples the issue of mental health law in community environments with the practical issues of service development in Africa.

### Mental Health Care Provision in Africa

As table 1 shows, the problem of limited resources is unavoidable in an African context. Per capita GDPs of less that US$2000 per year are common. While members of the African Union have affirmed their objective of spending fifteen per cent of their national budgets on health [3], many countries are unlikely to reach that target in the near future. Government per capita expenditures on health are often less than US$50 per year, and in some cases are less than US$10 per year. By comparison, governments of developed countries generally spend in the range of US$ 1500-2000 per capita per year on health. In Africa, health funding is often focused on a small number of national hospitals, leaving little for health care provision outside major urban centres. In Kenya, for example, the national hospitals in Nairobi consume 90% of the national health budget.

Mental health budgets in turn are a small part of the overall health budget. The share of mental health of the global burden of disease was roughly 12 per cent in 2000, expected to rise to 15 per cent by 2020 [4]. Health budgets devoted to mental health internationally often do not reach this proportion, but the proportions in Africa can be startlingly small - often less than one per cent of the health budget, and the overall health budget itself may only be around $10 per capita per year. In the context of such small health budgets overall, the result is miniscule funding actually available for mental health services. This is largely devoted to staff salaries and a small number of inpatient beds. Kenya for example has less than 1000 beds for a population of roughly 40 million, and of these, most are in Nairobi. Each province of around 4-5 million population has only 20 psychiatric inpatient beds. Each district of 250,000 has 1-2 psychiatric nurses, and less than 10% of districts have any inpatient psychiatric beds. In some African countries, there would appear to be virtually no state investment in specialist mental health services.

The result is minimal specialist mental health services. According to the WHO in 2005, (WHO, Mental Health Atlas, 2005) there were no psychiatrists in either Angola or Malawi, although Malawi has since successfully recruited a psychiatrist. On average, there is roughly one psychiatrist per million people in Africa, and this situation has been greatly aggravated by brain drain [5]. Kenya has 23 psychiatrists in the public service for 40 million population; Tanzania has 13 for 42 million. Psychiatric nurses, social workers and psychologists are in similarly short supply. Kenya has 250 psychiatric nurses deployed in psychiatry in the country, but the rate of production is far less than the rate of loss to retirement, mortality and brain drain (both overseas and internal). Indeed, in 2009, Kenya produced only one psychiatric nurse for the country. 12 were trained that year, but most were from other African countries, to which they returned. Numbers of Kenyan student psychiatric nurses fell in recent years since students now have to pay course fees, but in 2010 numbers are fortunately rising again.

These problems are exacerbated by geography. Outside RSA, the average area per psychiatrist in sub-Saharan Africa ranges from roughly 17,000 km^2 ^per psychiatrist in Swaziland, to 342,000 km^2 ^per psychiatrist in the Congo. To put that in context, the comparable numbers for Australia is 2600, the United States 230, and France and the UK are 40 km^2 ^per psychiatrist. Averages in this context must of course be approached with care. In practice, mental health professionals, and psychiatrists in particular, are likely to be concentrated in urban areas. For these urban populations, specialist services will be considerably more accessible than for people outside these urban areas. For people in rural areas, the concentration of specialist services in cities and the sparse coverage of primary care (1 clinic per 10,000 population) [6] means that the nearest medical facilities may be a very long way away indeed, a difficulty exacerbated by limited public transportation infrastructure.

Available treatments are limited. New generation anti-psychotics and antidepressants are unlikely to be available in the public sector because of price, and in any event do not achieve better outcomes although they do have better side effects. Older medicines are generally affordable by both governments and by clients if they have to cost share, but even these are often in short supply, and public distribution is often problematic because of difficulties in procurement from other countries, poor quality and lack of quality control of imported medications, and changes in distribution mechanisms, such as a shift in Kenya from a push system of drug kits for health facilities to a pull system of ordering by the health facilities. This has led not only to lack of psychotropics in health facilities but even of antimalarials. Training in new psychological therapies such as CBT is limited, and research suggests it needs continued supervision if its implementation is to be effective. Training may therefore be more effectively devoted to general psychosocial skills until such time as close supervision is sufficiently widespread to support implementation of CBT and other such specific therapies.

If primary care is to be a major provider of assessment and treatment of people with mental disorders, it is important that it receives regular support and supervision from the psychiatric nurses and others at the district level, and indeed that the districts receive regular support and supervision from the psychiatrists (if any) at the provincial level. However, shortage of specialists and lack of funds for transport means that, even where training for primary care and for district level staff is available, continuing supervision from the next level of care is difficult to sustain on a regular basis. Indeed in Kenya, under current health sector reform plans, primary care supervision for all subject areas is intended to be delivered by district public health nurses, who are the district cadre enabled to travel to primary care on a regular basis. Thus if the district public health nurses are to include mental health in their supervision activities, they will need to receive some training to support this role. This has now been delivered for 60 district public health nurses. Local inpatient provision for complex referrals who cannot be managed safely at home is similarly scarce (as mentioned above less than 10% districts in Kenya have any inpatient beds for psychiatric patients, and provincial hospitals serving 5 million population only have 20 beds each), and such facilities as there are may be understaffed.

Formalised specialist community support programmes are extremely rare, and indeed are logistically impractical and a misuse of scarce resource on a national basis, comparing numbers of potential clients with numbers of available specialist staff [7]. Thus if there is one psychiatric nurse per 250,000 population, he or she cannot deliver specialist community support to all who need it, but will instead need to see complex referrals and support primary care staff to see everyone else. Therefore there has long been recognition that in low resource settings especially, but also in richer countries, involvement of primary care is crucial and integration of mental health into primary care is essential for population access to mental health care [8].

The provision of health services is complicated by the existence of traditional healers, who are often the first port of call for the vast bulk of the population, including people who suffer from mental health problems. There has been little research on the effectiveness of indigenous treatments in terms of health and social outcomes, and it seems reasonable to speculate that their practices will vary considerably between regions, communities, and individual practitioners. There has certainly been documentation of harmful practices by some practitioners, but there is no reason to believe such practices are found in all healers. Traditional healers are often well embedded in the community, and are seen to understand the cultural and community context, and give time, and there are accounts of helpful synergistic work [9].

In the west, formal community mental health services are largely staffed by community based psychiatrists, psychiatric nurses, psychologists, occupational therapists and some less qualified but nonetheless paid care workers, delivering services collectively for around 10,000 population, in liaison with primary care services. In poor countries, these specialist staff are very scarce. One or two psychiatric nurses will be available at the district level, for a population of around 250,000-500,000. Therefore community mental health services in Africa need to be developed in very different ways to those in the west. Key elements will involve integration of mental health into primary care, so that it is fully integrated into the general work of the primary care health workers, and the linked volunteer community health workers. It is also worth noting that in the west, primary care is led by general practitioners, supported by practice nurses and others. In the UK there is one GP per 1700 clients. However in Africa, general practitioners are scarce and tend to be congregated in the private sector in the large cities, while most public primary care centres are staffed by nurses and clinical officers or medical assistants with a 3 year rather than six year medical training. The primary care nurse and clinical officer will usually be responsible for 10,000 population or more.

Careful liaison with THPs may extend the health human resource available for people with mental disorders. Mental health is not just a health issue, it also links to the other sectors of employment, education, social welfare, criminal justice system etc Therefore the primary care team will also need to make such collaborative local linkages with the other local sectors in order to promote mental health, prevent illness and address mental disorders. Such local intersectoral linkages will be more effective if they are supported by intersectoral management teams at the municipal, provincial and national levels [10].

Legislative provision similarly tends toward the rudimentary. While more modern mental health statutes are starting to appear in Africa [11,12], in many countries the law remains as it was inherited from colonial administrations. Frequently it does not reflect current international legal norms; often there appear to be no mechanisms such as regular continuing professional development, structures of administrative audit and oversight and review boards to ensure its implementation and enforcement, resulting in limited if any application. These problems also arise in countries where there are new statutes. Kenya passed new mental health legislation in 1989, but as is often the case, there has been incomplete implementation as a result of a lack of funds to train health and social care staff, police, prison staff and others charged with its implementation. Such lack of funds for continuing professional development of staff is a significant difficulty faced by African countries, and needs to be taken into account when new legislation is contemplated.

The picture, therefore, is complex. Economic resources are limited, and limited personnel and capital infrastructure will for the foreseeable future impose significant restrictions on service provision. What role, if any, is there for mental health law in this situation?

### The Role of Law in the Community

The movement towards community-based service provision has been given a boost internationally by the introduction of the CRPD. Previous international instruments such as the United Nations Mental Illness Principles of 1991 had started from the premise that control of people with mental disabilities was appropriate in some circumstances; the issue was the appropriate scope of that control. The CRPD, by comparison, starts from the premise that people with disabilities, including those with mental disabilities, have the same human rights as everyone else in society, and emphasises principles including non-discrimination, enhancement of autonomy, equality of opportunity and, perhaps most significantly for the current context, full and effective participation and inclusion in society. The CRPD further reflects a political shift: where in the negotiation of previous international instruments, service users and their organisations were at the edges of the process, if they were consulted at all, for the CRPD they were key players, involved throughout the negotiations. Further, thanks to the generosity primarily of the Swedish government, service users from relatively poor countries were included, including a number from Africa. The result is that the CRPD has considerable buy-in from these stakeholders and their organisations, and the Convention itself requires the continued involvement of disabled peoples' organisations in the development of policy and the implementation of the Convention. As of August 2010, 146 countries had signed the Convention, including 89 which had ratified it. In Africa, 38 countries had signed the Convention and 23 ratified it as of that date [13].

The CRPD provides a broad array of rights that will be relevant to community services. These include rights to independent living and to inclusion in the community and personal mobility (art 20), to privacy (art 22), to the home and to family life, including reproductive rights (art 23), to education (art 24), to work and employment (art 27), to an adequate standard of living and social protection (art 28), to participation in public and political live (art 29), and to participation in culture, recreation, leisure activities and sports (art 30). Many of these will attack determinant factors of mental ill health, and should therefore be supported by mental health professionals. It also provides a right to 'the enjoyment of the highest attainable standard of health without discrimination on the basis of disability' (art 25), and new rights concerning the exercise of legal capacity (art 12).

Certainly, programmes to advance these values will be beneficial to people with mental disabilities. While rights to medical treatment are included, refreshingly, the CRPD looks beyond the provision of medical care to standards of living, presumably including issues of diet, hygiene and shelter - no doubt potentially beneficial factors for promotion of mental health and reduction of mental illness. But will law really help to advance these matters? If there is a social consensus favouring good services coupled with financial resources and political will, the counterargument goes, then good services will be provided; if there is no such consensus, it is unlikely that such services will appear, whatever the law may say. What exactly can law bring?

Certainly there is merit in that view. Many of the rights identified above are already contained in other binding treaties, with at best limited results in many countries. While the articulations of the rights in the CRPD is particularly detailed and is targeted at persons with disabilities, passing a convention is not the same as seeing substantive change. This is a salient reminder that law can be at best part of the way forward: education and political action are equally vital part of the road to change. That said, the argument can be overstated. Law has a symbolic as well as a functional role, and can normalise values over time, giving new ideas the gravitas of respectability. The CRPD's articulation of pre-existing rights expressly in the context of people with disabilities, and its placement of these rights in a community context is thus potentially significant,

Further, the CRPD, unlike some previous international conventions, does have enforcement procedures built in. A UN Committee is being established to which countries will be obliged to report, and which will in turn publish public reports relating to compliance with the CRPD. For countries signing the optional protocol to the CRPD, the committee will also judge complaints made by individuals regarding alleged violations of the convention. While the decisions of that committee may not necessarily be enforceable in domestic courts, their visibility will create moral pressures to comply with the convention. Further, the convention requires each country to establish its own implementation and enforcement mechanisms (art 33). Failure to do so is likely to draw the attention of the committee, so again, pressures toward implementation are part of the framework of the convention.

More problematic is what, in substantive terms, an African government should do. In some cases, the CRPD is at least in principle fairly clear. Some of its rights are immediately realisable, and governments are under an immediate obligation to comply with them. By way of example, the CRPD establishes a new set of relations concerning people of limited capacity and the state. Plenary guardianship, by which an individual with mental disability is deprived of large swathes of decision-making authority (and often also apparently unrelated rights, such as the right to vote or the right to work), often on minimal evidence, is not consistent with the CRPD provisions. Instead, the CRPD requires the introduction of supported decision-making, ensuring that the individual who is capable of making a choice is given the authority to make that choice. Restrictions on that authority may be made only in the face of clear evidence relating to the specific decision in question. For much of the world, including much of Africa, this will involve a significant change of approach. Existing guardianship regimes are contained in law, and the new provisions will need to have statutory form as well.

Other changes will be more programmatic, and extend over time. As regards the right to health, for example, it does seem that localised solutions, as far as possible keeping individuals in their local communities are to be preferred, and hospitalisation in distant institutions is to be avoided, for both human and financial reasons. The implementation of programmes to implement these solutions will take time, however. This is not necessarily a problem for the CRPD, as the right to health, like most of the other social rights noted in the opening paragraph of this section, are subject to 'progressive realisation': it is acknowledged that it may take time to bring them about [14]. This should not be understood, as it occasionally seems to be, as an excuse for governments to do nothing. Rights subject to progressive realisation are still rights in international law, and national governments are under an immediate duty to work towards their realisation with due dispatch. Governments are therefore under a current obligation to look at overall budget allocations to determine whether they are providing appropriate funding to health, and are required to look within health budgets to ensure that they are providing the best outcomes for the money and personnel available. The non-discrimination provisions of the CRPD contained in article 5 and the individual substantive articles make it clear that people with disabilities (including mental disabilities) have as much of a right to health, education and other services as anyone else in the community, suggesting some re-balancing of health budgets may be required. Progressive realisation does acknowledge however that implementing those decisions may involve training people and introducing new programmes, and that such implementation can take time.

Perhaps more important, progressive realisation creates an ongoing duty to re-visit services, improving them as financial, political, scientific and social circumstances permit. The best attainable standard of health is thus a moving target that takes into account improvements in financial resources, professional developments, and drug and other therapies.

What is the role of domestic law in this process? Certainly, individual programmes or law reforms will need to be given shape in domestic legislation: if the decision is made to provide a new form of benefit to people with mental disabilities in partial compliance with the right to an adequate standard of living for persons with disabilities (art 28), that new benefit will almost certainly need statutory structure to mandate its introduction. It is law that creates the legal authority for such programmes.

This takes a static view of services, however, where the real issue may well be the need to put in place an ongoing programme of reforms that will develop over time. Domestic law is often less successful at embodying such aspirational changes, as such changes are often dependant on financial and other resources which the law does not of itself create. What law can do is to create advocacy bodies - 'mental health commissions' for want of a better phrase - to foster development of the programmatic agenda over time, bodies that ensure that the relevant reforms do not slip off the political agenda. To be successful, these need to be staffed by competent people who are independent of government, but who will have the respect both of government and the relevant civil society stakeholders: when they make recommendations, they must have the gravitas that the government will have to engage with them. They need to be given access to the information that is relevant to allow them to comment meaningfully on the progress being made by government in reaching the objectives of the progressively realisable rights. And they must report publicly, to ensure that pressure remains on the government to make progress in these areas. International law requires an independent inspection body to ensure appropriate standards in psychiatric hospitals and other places of detention; it may well be sensible that the body that comments on progress towards the progressively realisable rights is also the body that performs such inspections.

Such bodies are likely to prove remarkably beneficial in an African context. Many of the systems of care that are provided will be culture-specific: it is not likely to be sensible or successful simply to transplant care models from Europe or America to Africa. It is not merely that the financial resources are not available. It is also that culturally and politically Africa is not western Europe or America, and it would be foolhardy to pretend otherwise. This is not merely a question of the structures of medical care. Communities and family structures may function very differently in Africa than elsewhere (and indeed may function differently in different parts of Africa). Priorities of service users and other stakeholders may differ depending on the locality, and fundamental questions of dignity may perhaps be articulated quite differently be service users in an African context than elsewhere: we will not really know until those discussions happen at the local level. What mental health commissions can do is to give serious consideration to the local situation, taking into account the relevant resources, available personnel, local priorities and values, and comment visibly on progress (or lack of progress) towards community integration and community services. Such bodies do of course have resource requirements - they must meet and travel, and the members will probably need to be paid for their time, and all that costs some money. The financial costs are not prohibitive, however, and if these bodies can develop appropriate strategies for mental health in Africa, they will be well worth the expense.

While an argument can be made that progress can be made with a limited budget, the issue of financial resources is unavoidable. It is difficult to see that many of the countries in Africa will be able to make significant progress without ongoing and significant financial support from international donors. What is required is not (or at least, not merely) short-term funding for pilot projects, but a real and ongoing commitment to funding to enable the establishment of sustained integrated health programmes that include population access to mental health care. The ideal may be for the design of programmes that African countries can pay for out of their own tax based budgets, but there is no realistic prospect that this ideal will be achieved any time soon. The failure of western governments to engage with this human right to health issue as it relates to access to mental health care is likely to preclude even the substantial improvements that can be made with relatively little money, because in so many African countries even these small sums are not actually available in the public sector. This returns the debate to an earlier theme: law has its role to play, but it is only part of a broader political and economic debate; and that debate must be had internationally as well as in the African domestic context.

## Conclusions

Mental health law is in a period of change. The movements to community care that have formed the direction of service provision for much of the last quarter century are now starting to be reflected in international law, with the CRPD serving as an important marker of the new approach. This does not mean that domestic law will cease to be relevant, but it does mean that new forms of domestic law will start to appear. Domestic law regarding abuse of human rights in Kenya has proved very useful for police to take action with THPs who are beating patients, for example.

Africa provides interesting potential for these changes. Unlike so many other places in the world, it does not have a strong tradition of institutionalisation, so there is less to unlearn and fewer large institutions to scale down. Equally, however, there is much that needs to be done to enable population access to mental health care via primary care and decentralised district level specialist services, with especial attention to building capacity in primary care staff, district supervisors and health management teams responsible for local planning. International experts may offer support in these endeavours, but the solutions must be tailored to take account of African culture, context and resources. The objective must be to ensure sustainable long-term development of suitable local services in African communities. To this end, domestic law has a role to play, especially if its central tenets can be integrated into basic and continuing training programmes for relevant sectors.

## Competing interests

The authors declare that they have no competing interests.

## Authors' contributions

PB was primarily responsible for the legal material in the article, compiled the information in Table 1, and contributed to the discussion of the African situation from the perspective of work in Lesotho. RJ was primarily responsible for the discussion of the medical material in the article, and in particular the material relating to East Africa. DK provided comments, particularly from the perspective of East Africa.

## Supplementary Material

Additional file 1 **Africa Table 1**. Basic Demographic Data regarding African Mental Health Provision, with comparators, 2005. Click here for file

## References

[B1] Jenkins R Kiima D Okonji M Njenga F Kingora J Lock S Integration of mental health into primary care and community health working in Kenya: context, rationale, coverage and sustainability Mental Health in Family Medicine 2010 7 37 47 PMC2925163 22477921

[B2] Jenkins R Kiima D Njenga F Okonji M Kingora J Kathuku D Lock S Integration of mental health into primary care in Kenya World Psychiatry 2010 9 118 120 20671901 10.1002/j.2051-5545.2010.tb00289.x PMC2911092

[B3] It's how you spend the money that saves lives http://www.irinnews.org/Report.aspx?ReportId=90000 IRIN News, 28 July 2010, accessed 12 August 2010.

[B4] World Health Organization The World Health Report 2001: Mental Health: New Understanding, New Hope 2001 Geneva: WHO

[B5] Jenkins R Gureje O Mullen P Kydd R Hatcher S Thompson K Carroll C Wong ML Hollins S International migration of psychiatrists PLOS One 2010 http://dx.plos.org/10.1371/journal.pone.0009049

[B6] Kiima D Jenkins R Mental health policy in Kenya-an integrated approach to scaling up equitable care for poor populations International Journal of Mental Health Systems 2010 4 1 19 10.1186/1752-4458-4-19 20584266 PMC2907308

[B7] Jenkins R Mbatia J Singleton N White B Prevalence of psychotic symptoms and their risk factors in urban Tanzania International Journal of Environmental Research and Public Health 2010 7 2514 1525 10.3390/ijerph7062514 20644687 PMC2905564

[B8] World Health Organization Report of the International Conference on primary health care 2010 Alma Ata, USSR. Geneva: WHO

[B9] Bartlett P McSherry B, Weller P Thinking About the Rest of the World: Mental Health and Rights Outside the 'First World' Re-Thinking Rights-Based Mental Health Law 2010 Oxford: Hart 397 418 http://eprints.nottingham.ac.uk/1415/

[B10] Jenkins R McCulloch A Friedli L Parker C Developing Mental Health Policy 2002 Psychology Press, Taylor and Francis Group Maudsley Monograph 4

[B11] Mental Health Care Act, Act No 17, 2002 (South Africa)

[B12] Mental Health Act, Act No 21, 1008 (Tanzania)

[B13] United Nations Convention on the Rights of Persons with Disabilities General Assembly A/61/611, 6 December 2006

[B14] Hunt P Report of the Special Rapporteur on the right of everyone to the enjoyment of the highest attainable standard of physical and mental health 2005 UN E/CN.4/2005/51. New York: Economic and Social Council, Commissioner of Human Rights

