# Supplementary material for: Mental health law in the community: thinking about Africa
Source: Int J Ment Health Syst. 2011 Sep 13;5:21. doi: 10.1186/1752-4458-5-21 (PMC3189124; doi:10.1186/1752-4458-5-21)
Supplement: Additional file 1 — Africa Table 1. Basic Demographic Data regarding African Mental Health Provision, with comparators, 2005. [file 1752-4458-5-21-S1.DOC]

Table I: Basic Demographic Data regarding African Mental Health Provision, with comparators, 2005

|  | **Area**  **(K km^2)^** | **Popula-tion (million)** | **Per Capita GDP**  **$US** | **Health Budget as % of GDP** | **Per Capita Expenditure on Health, Total /Govt US$** | **MH Budget as % of Health Budget** | **Per 10,000 population** | | | | **Per 100,000 population** | | | |
| --- | --- | --- | --- | --- | --- | --- | --- | --- | --- | --- | --- | --- | --- | --- |
|  |  |  |  |  |  |  | **MH Beds (Total)** | **MH Beds in MH Hospitals** | **MH Beds in General Hospitals** | **MH Beds in other settings** | **Number Psychiatrists** | **Number of MH Nurses** | **Number psychologists** | **Number MH social workers** |
| **Africa (selection)** |  |  |  |  |  |  |  |  |  |  |  |  |  |  |
|  |  |  |  |  |  |  |  |  |  |  |  |  |  |  |
| **Algeria** | 2381 | 35 | 7000 | 4.1 | 169/127 | N/A | 1.4 | .86 | .2 | .36 | 1.1 | 1.1 | .8 | 0 |
| **Angola** | 1247 | 13 | 8900 | 4.4 | 70/44 | 0 | .13 | .7 | .6 | 0 | 0 | 0 | 0 | 0 |
| **Benin** | 112 | 9 | 1500 | 4.4 | 39/18 | N/A | .08 | 0 | .03 | .05 | 1.2 | 0 | .05 | .02 |
| **Botswana** | 582 | 2 | 13000 | 6.6 | 381/252 | 1 | 1.1 | .7 | .4 | 0 | .4 | 9 | .3 | 3 |
| **Burkina Faso** | 274 | 16 | 1200 | 3.0 | 27/16 | N/A | .18 | .06 | .12 | 0 | .05 | .4 | .03 | .02 |
| **Burundi** | 27 | 10 | 300 | 3.6 | 19/11 | N/A | .1 | .1 | 0 | 0 | .02 | 0 | .2 | 1.5 |
| **Cameroon** | 475 | 19 | 2300 | 11.8 | 42/16 | .1 | .08 | .07 | .0007 | 0 | .03 | .2 | 0 | .1 |
| **Central African Republic** | 623 | 5 | 700 | 4.5 | 58/30 | N/A | .07 | 0 | .07 | 0 | .03 | .03 | .08 | .03 |
| **Chad** | 1284 | 11 | 1600 | 2.6 | 17/13 | N/A | .02 | .01 | .01 | 0 | .01 | .01 | .01 | 0 |
| **Congo** | 342 | 4 | 4100 | 2.1 | 22/14 | N/A | .06 | - | .06 | - | .03 | .1 | .26 | N/A |
| **Côte d’Ivoire** | 322 | 21 | 1700 | 6.2 | 127/20 | .01 | .15 | .13 | .02 | 0 | .2 | .2 | .07 | .03 |
| **Djibouti** | 23 | .7 | 2800 | 7.0 | 90/53 | N/A | .07 | 0 | .07 | 0 | 0 | .16 | 0 | 0 |
| **DR Congo** | 2345 | 71 | 300 | 3.5 | 12/5 | N/A | .17 | .15 | .009 | .009 | .04 | .03 | .01 | .4 |
| **Egypt** | 1001 | 80 | 6000 | 3.9 | 153/75 | 9 | 1.3 | 1.1 | .1 | .1 | .9 | 2 | .4 | .1 |
| **Ethiopia** | 1104 | 88 | 900 | 3.6 | 14/6 | N/A | .07 | .06 | .01 | 0 | .02 | .3 | .08 | .08 |
| **Gabon** | 268 | 2 | 13900 | 3.6 | 197/94 | .3 | .7 | .6 | .06 | 0 | .3 | 1 | .5 | 2 |
| **Gambia, The** | 11 | 2 | 1400 | 6.4 | 78/39 | N/A | .78 | .78 | 0 | 0 | .08 | 0 | 0 | .08 |
| **Ghana** | 239 | 24 | 1500 | 4.7 | 60/36 | .5 | 1.03 | 1 | .01 | .02 | .08 | 2 | .04 | .03 |
| **Guinea** | 246 | 10 | 1000 | 3.5 | 61/33 | N/A | .05 | .05 | 0 | 0 | .04 | 0 | 0 | 0 |
| **Kenya** | 580 | 40 | 1600 | 7.8 | 114/24 | .01 | .4 | .3 | .05 | .02 | .2 | 2 | .01 | .2 |
| **Lesotho** | 30 | 2 | 1700 | 5.5 | 101/80 | 7 | .8 | .3 | .5 | 0 | .05 | .2 | .09 | 1.2 |
| **Liberia** | 111 | 4 | 400 | 4.3 | 127/97 | N/A | .08 | .08 | 0 | 0 | .03 | .03 | 0 | 0 |
| **Libya** | 1760 | 6 | 15000 | 2.9 | 239/134 | N/A | 1 | 1 | 0 | 0 | .18 | .5 | 5 | 1.5 |
| **Madagascar** | 587 | 21 | 1000 | 2.0 | 20/13 | .82 | .17 | .08 | .08 | .01 | .08 | .3 | .03 | .02 |
| **Malawi** | 118 | 15 | 900 | 7.8 | 39/14 | 2 | .37 | N/a | N/a | N/a | 0 | 2.5 | 0 | 0 |
| **Mali** | 1240 | 14 | 1200 | 4.3 | 30/12 | .02 | .2 | 0 | .1 | .05 | .06 | .15 | .02 | .01 |
| **Mauritius** | 2 | 1 | 12400 | 3.4 | 323/192 | .3 | 9.5 | 8 | 1 | .5 | 1 | 5 | 1 | 1 |
| **Morocco** | 447 | 32 | 4600 | 5.1 | 199/78 | N/A | .783 | .52 | .17 | .1 | .4 | 2.2 | .03 | .007 |
| **Mozambique** | 799 | 22 | 900 | 5.9 | 47/32 | N/A | .23 | .2 | .04 | .01 | .04 | .01 | .05 | .01 |
| **Namibia** | 824 | 2 | 6400 | 7.0 | 342/232 | N/A | 1.5 | 1.5 | 0 | 0 | .2 | 0 | 6 | 6 |
| **Niger** | 1267 | 16 | 700 | 3.7 | 22/9 | N/A | .2 | 0 | .2 | 0 | .04 | .04 | .03 | .05 |
| **Nigeria** | 924 | 152 | 2400 | 3.4 | 31/7 | N/A | .4 | .3 | .04 | .01 | .09 | 4 | .02 | .02 |
| **Rwanda** | 26 | 11 | 900 | 5.5 | 44/24 | 1 | .2 | .2 | 0 | 0 | .03 | .8 | .3 | 0 |
| **Senegal** | 197 | 14 | 1600 | 4.8 | 63/37 | 9 | .3 | .15 | .1 | .05 | .16 | .06 | .04 | .035 |
| **Sierra Leon** | 71 | 5 | 900 | 4.3 | 26/16 | N/A | .47 | .32 | .11 | .03 | .02 | .04 | 0 | .06 |
| **South Africa** | 1219 | 49 | 10000 | 8.6 | 652/270 | N/A | 4.5 | 4 | .38 | .12 | 1.2 | 7.5 | 4 | 20 |
| **Sudan** | 2505 | 42 | 2300 | 3.5 | 39/7 | N/A | .2 | .18 | .2 | 0 | .09 | .2 | .17 | .1 |
| **Swaziland** | 17 | 1 | 4400 | 3.3 | 167/115 | .3 | 2 | 2 | 0 | 0 | .1 | 10 | .1 | .1 |
| **Tanzania** | 947 | 42 | 1400 | 4.4 | 26/12 | 7 | .7 | .36 | .04 | .3 | .04 | 2 | .005 | .2 |
| **Togo** | 57 | 6 | 900 | 2.8 | 45/22 | .2 | .4 | .3 | .1 | 0 | .04 | 0 | .02 | 0 |
| **Tunisia** | 164 | 11 | 8000 | 6.4 | 463/350 | N/A | 1.13 | .85 | .27 | 0 | 1.6 | .2 | .6 | N/A |
| **Uganda** | 241 | 33 | 1300 | 5.9 | 57/33 | .7 | .44 | .22 | .22 | .009 | 1.6 | 2 | 2 | 2 |
| **Zambia** | 753 | 12 | 1500 | 5.7 | 52/27 | 0 | .5 | .17 | .18 | .07 | .02 | 5 | .04 | .04 |
|  |  |  |  |  |  |  |  |  |  |  |  |  |  |  |
| **Comparitors** |  |  |  |  |  |  |  |  |  |  |  |  |  |  |
| **Australia** | 7741 | 22 | 38800 | 9.2 | 2532/  1718 | 9.6 | 3.9 | 1.2 | 2.7 | 1 | 14 | 53 | 5 | 5 |
| **Czech Rep.** | 79 | 10 | 25000 | 7.4 | 1139/  1031 | 3 | 11.4 | 9.8 | 1.5 | .2 | 12.1 | 33 | 4.9 | N/A |
| **France** | 643 | 64 | 33000 | 9.6 | 2567/  1951 | 8 | 12 | 7 | 3 | 2 | 22 | 98 | 5 | N/a |
| **Hungary** | 93 | 10 | 18600 | 6.8 | 914/686 | 8 | 9.6 | 2.3 | 7.2 | .1 | 9 | 19 | 2 | 1 |
| **UK** | 244 | 61 | 35000 | 5.8 | 1989/  1634 | 10 | 5.8 | N/a | N/a | N/a | 11 | 104 | 9 | 58 |
| **US** | 9827 | 310 | 46400 | 13.9 | 4887/  2168 | 6 | 7.7 | 3.1 | 1.3 | 3.3 | 13.7 | 6.5 | 31.1 | 35.3 |
|  |  |  |  |  |  |  |  |  |  |  |  |  |  |  |

Sources for data columns 1-3: Central Intelligence Agency, *World Fact Book*, (Washington: CIA, 2009). Source for data columns 4-13: World Health Organization, *Mental Health Atlas 2005* (Geneva: World Health Organization, 2005).
